# Small angle X-ray scattering studies of CTNNBL1 dimerization and CTNNBL1/CDC5L complex

**DOI:** 10.1038/srep14251

**Published:** 2015-09-18

**Authors:** Jae-Woo Ahn, Kyeong Sik Jin, Hyeoncheol Francis Son, Jeong Ho Chang, Kyung-Jin Kim

**Affiliations:** 1 School of Life Sciences, KNU Creative BioResearch Group, Kyungpook National University, Daehak-ro 80, Buk-ku, Daegu 702-701, Korea; 2 Pohang Accelerator Laboratory, Pohang University of Science and Technology, Jigok-ro 80, Pohang, Kyungbuk 790-784, Korea; 3 Department of Biology, Teachers College, Kyungpook National University, Daehak-ro 80, Buk-ku, Daegu 702-701, Korea

## Abstract

The hPrp19/CDC5L complex is a non-snRNP spliceosome complex that plays a key role in the spliceosome activation during pre-mRNA splicing, and CTNNBL1 and CDC5L are essential components of the complex. In this study, to investigate the oligomeric state of CTNNBL1 in solution, we performed small angle X-ray scattering experiments in various concentrations of NaCl. We observed that CTNNBL1 existed as a dimer in physiological NaCl concentrations. Site-directed mutagenesis experiment of CTNNBL1 confirmed that N-terminal capping region and the first four ARM repeats are important for dimerization of the protein. We also found that the positively-charged NLS3-containing region (residues 197–235) of CDC5L bound to the negatively-charged patch of CTNNBL1 and that the CTNNBL1/CDC5L complex formed a heterotetramer consisting of one CTNNBL1 dimer and one CDC5L dimer. Moreover, reconstruction of 3D models of CTNNBL1/CDC5L complexes containing CTNNBL1 and three different truncated forms of CDC5L showed that the CDC5L^141–196^ region and the CDC5L^236–377^ region were positioned at the top of the N-terminal capping region and at the bottom of ARM VII of CTNNBL1, respectively.

Pre-mRNA splicing is a modification process that converts nascent pre-mRNA transcripts into mature mRNA transcripts by removing introns and relinking exons. The process is catalyzed by the spliceosome, a dynamic megadalton-sized complex consisting of four ribonucleoprotein particles (snRNPs; U1, U2, U5, and U4/U6) and non-snRNP complexes such as the hPrp19/CDC5L complex^1^,^2^,^3^. snRNPs and non-snRNP proteins assemble in consecutive order through a highly dynamic process to form the spliceosome^1^,^4^,^5^. Various non-snRNP complexes are involved and are known to play crucial roles in spliceosome formation and the splicing reaction. One well-known non-snRNP complex is the human hPrp19/CDC5L complex, which is referred to as the nineteen complex (NTC) in yeast^6^,^7^. The hPrp19/CDC5L complex plays a crucial role in spliceosome activation by participating in snRNP rearrangement^3^.

The hPrp19/CDC5L complex is composed of seven components: hPrp19, CDC5L, PRL1, AD002, SPF27, CTNNBL1, and HSP73^8^. Homologues of hPrp19, CDC5L, PRL1, and SPF27 in the hPrp19/CDC5L complex have been identified in the yeast NTC, whereas CTNNBL1, HSP73, and AD002 are only found in the hPrp19/CDC5L complex^6^,^9^,^10^. Interaction studies of the hPrp19/CDC5L complex have shown that CTNNBL1 interacts with CDC5L and AD002. Limited proteolysis of the purified hPrp19/CDC5L complex has suggested that the CTNNBL1/AD002 heterocomplex associates with the core of the hPrp19/CDC5L complex by interacting with the N-terminal domain of CDC5L^11^. In addition to functioning in spliceosome activation, some of the hPrp19/CDC5L complex components participate in other cellular processes. CDC5L regulates RNA processing and mitotic entry in the cell cycle, depending on its phosphorylation state^12^,^13^. CDC5L also contributes to gene regulation by binding to a site-specific DNA sequence^14^. CTNNBL1 interacts with activation-induced deaminase (AID), suggesting it has a role in antibody diversity^15^. Moreover, CTNNBL1 deficiency results in embryonic lethality in mice^16^, and a genome-wide survey and functional brain imaging study have identified CTNNBL1 as a memory-related protein^17^.

Recently, our group determined the crystal structure of CTNNBL1, which revealed that the protein has an ARM-repeat protein fold. CTNNBL1 formed a dimer in 200 mM NaCl, unlike other canonical ARM-repeat proteins such as β-catenin and importin-α. In addition, site-directed mutagenesis and pull-down assays showed that the positively-charged N-terminal domain of CDC5L bound to the negatively-charged patch of CTNNBL1, just as nuclear localization sequences (NLSs) bind to importin-α, with a stoichiometry of 2:2. However, another crystal structure of CTNNBL1, reported at the same time, showed that the protein did not form a dimer, but rather functioned as a monomer^18^,^19^,^20^. Because the oligomeric status of CTNNBL1 is the basis for understanding the molecular architecture of the hPrp19/CDC5L complex, the controversy surrounding the oligomeric status of CTNNBL1 needs to be resolved. In this report, we generated *De novo* models of CTNNBL1 in various concentrations of NaCl using small-angle X-ray scattering (SAXS) experiments. We found that the protein formed a stable dimer in solution. We also constructed a *De novo* model of the CTNNBL1/CDC5L binary complex, which might provide a structural basis for the molecular architecture of the hCTNNBL1/CDC5L complex.

## Results

### Dimeric state of CTNNBL1 in solution

**Figure 1:**
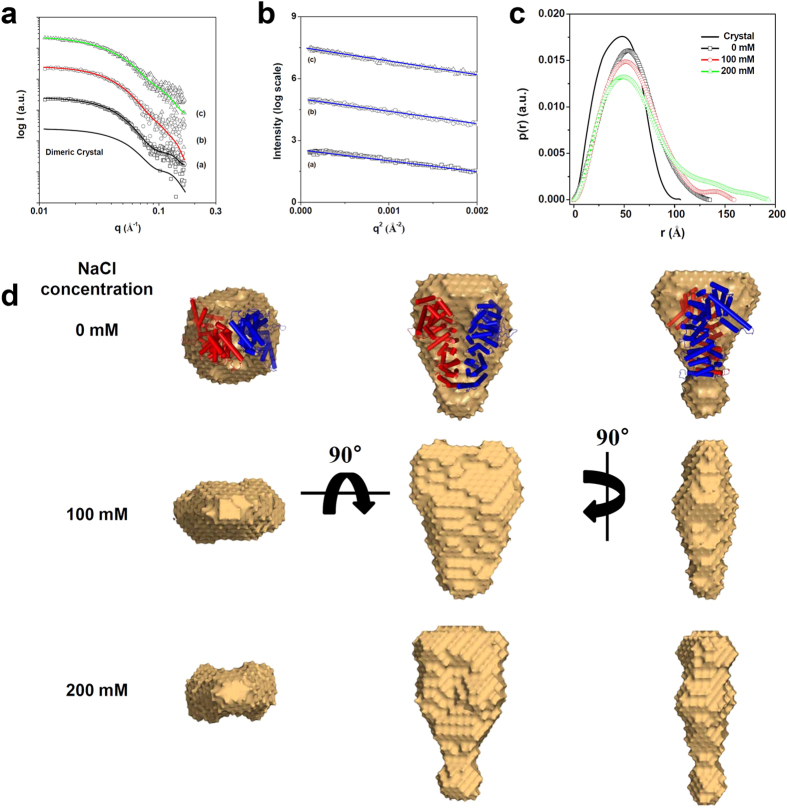
SAXS measurement of CTNNBL1. (**a**) X-ray scattering profiles of CTNNBL1 proteins in various concentrations of NaCl. The open symbols are the experimental data, and the solid lines are the X-ray scattering profiles obtained from the dummy atom models with the lowest χ = 1.560–1.640 values by the program DAMMIF. The solid line without symbols is the theoretical SAXS curve calculated from the crystal structure of CTNNBL1. a, b, and c indicate the X-ray scattering profiles of CTNNBL1 in 0, 100, and 200 mM NaCl, respectively. For clarity, each plot is shifted along the log *I*(*q*) axis. (**b**) Guinier plots of the X-ray scattering profiles of CTNNBL1 proteins. The straight lines were obtained from the linear regression of the scattering data in the *q*^2^ region. a, b, and c indicate the X-ray scattering profiles of CTNNBL1 in 0, 100, and 200 mM NaCl, respectively. For clarity, each plot is shifted along the ln *I*(*q*) axis. (**c**) Pair distance distribution functions *p*(*r*) for CTNNBL1 protein. The *p*(*r*) functions of CTNNBL1 in solutions with various concentrations of NaCl were calculated using the program GNOM. The NaCl concentrations used are indicated. (**d**) Reconstructed structural models of CTNNBL1 protein. For each reconstruction, five independent models were generated, compared, and averaged (Mean values of NSD of reconstructed models =0.493–0.650). The filtered model was calculated using the program DAMAVER.

To investigate the oligomeric state of CTNNBL1 in solution and provide a structural platform for deciphering the overall structure of the CTNNBL1/CDC5L complex, synchrotron SAXS measurements were carried out in a wide range of NaCl concentrations (0, 100, and 200 mM) (Fig. 1a). The calculation of the radius of gyration (*R*_g,G_) and the molecular mass (MM) from the X-ray scattering data showed that the molecular weight of CTNNBL1 in 0–200 mM NaCl solution was ~120 kDa (Table 1, Fig. 1a). These results indicate that CTNNBL1 exists in a dimeric state under NaCl concentrations lower than 200 mM. Straight lines were observed in the Guinier analysis using small *q* regions of the SAXS measurements, indicating that CTNNBL1 exists as monodisperse states with a dimeric conformation (Fig. 1b). The oligomeric states of CTNNBL1 under different NaCl concentration were further confirmed by pair distance distribution function (*p*(*r*) function) calculation. The *p*(*r*) function for CTNNBL1 in all NaCl concentrations clearly displayed a unimodal symmetrical peak pattern as a single symmetrical peak pattern calculated from the homodimeric crystal structure (Fig. 1c).

We then reconstructed model-independent structural models to obtain a 3D representation of the structure of CTNNBL1. The conformation of CTNNBL1 in 0 mM NaCl solution was similar to that observed in the homodimeric CTNNBL1 crystal structure (Fig. 1d). However, the structural models of CTNNBL1 in solutions of 100 and 200 mM NaCl exhibited dissimilarities when compared to the model of CTNNBL1 in 0 mM NaCl, although the former adopted homodimeric states as well. In the reconstructed model of CTNNBL1 in 100 mM NaCl, the protein was somewhat extended along the vertical axis with its thickness reduced; in 200 mM NaCl, the protein was markedly extended along the vertical axis (Fig. 1d). These solution models are consistent with the results of the *p*(*r*) function analysis (Table 1). Taken together, we propose that CTNNBL1 exist as a dimer under physiological NaCl concentrations.

### Dimerization interface of CTNNBL1

**Figure 2:**
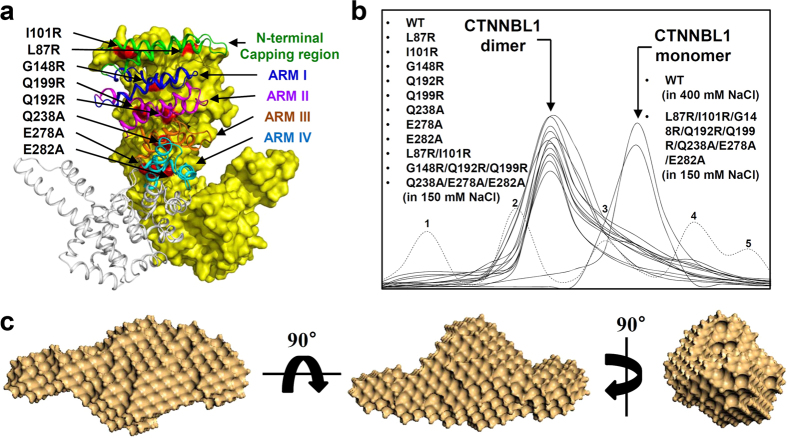
Dimerization of CTNNBL1. (**a**) Interface of CTNNBL1 dimerization. The CTNNBL1 dimer is shown. One monomer is shown as a surface model in yellow, and the residues modified by site-directed mutagenesis are shown in red and labeled appropriately. The other monomer is shown as a cartoon diagram. The N-terminal capping region, ARM I, ARM II, ARM III, and ARM IV are shown in green, blue, magenta, orange, and cyan, respectively, and labeled appropriately. (**b**) Size-exclusion chromatography of the CTNNBL1 mutants. The elution peaks corresponding to the dimeric or monomeric form of CTNNBL1 are indicated by arrows. The CTNNBL1 mutants L87R, I101R, G148R, Q192R, Q199R, Q238R, E278A, E282A, L87R/I101R, G148R/Q192R/Q199R, and Q238A/E278A/E282A eluted as dimers at 150 mM NaCl, with molecular weights of ~110 kDa. The elution peak was compared with that of wild-type CTNNBL1 at 150 mM NaCl. The CTNNBL1 eight-point mutant (L87R/I101R/G148R/Q192R/Q199R/Q238A/E278A/E282A) eluted as a monomer at 150 mM NaCl, with a molecular weight of ~55 kDa. The elution peak was compared with that of wild-type CTNNBL1 at 400 mM mM NaCl. For a precise analysis of molecular weight, standard samples of ferritin (440 kDa), aldolase (158 kDa), conalbumin (75 kDa), ovalbumin (44 kDa), and carbonic anhydrase (29 kDa) were used for calibration in size-exclusion chromatography experiments; the peaks are labeled from 1 to 5, respectively. (**c**) Reconstructed structural model of CTNNBL1 mutant. For the reconstruction, five independent models were generated, compared, and averaged (Mean values of NSD of reconstructed models = 0.493–0.650). The filtered model was calculated using the program DAMAVER.

Our crystal structure of CTNNBL1 showed that CTNNBL1 form a dimer. We suggested that the N-terminal capping region and the first four ARM repeats (ARM I, ARM II, ARM III, and ARM IV) participate in the dimerization of CTNNBL1^18^. To confirm the dimerization of CTNNBL1 and identify its dimerization interface, we performed site-directed mutagenesis experiments. First, we mutated eight residues located at the suggested dimerization interface and generated the single-point mutants CTNNBL1^L87R^, CTNNBL1^I101R^, CTNNBL1^G148R^, CTNNBL1^Q192R^, CTNNBL1^Q199R^, CTNNBL1^Q238A^, CTNNBL1^E278A^, and CTNNBL1^E282A^ (Fig. 2a). When the mutant proteins were assessed with size-exclusion chromatography, all eight mutants were eluted in a dimeric state with a molecular weight of ~110 kDa (Fig. 2b). We then generated several double- or triple-point mutants, CTNNBL1^L87R/I101R^ (capping region), CTNNBL1^G148R/Q192R/Q199R^ (ARM I and ARM II), and CTNNBL1^Q238A/E278A/E282A^ (Arm III and Arm IV), and analyzed them with size-exclusion chromatography. However, these mutant proteins were also eluted in a dimeric state with a molecular weight of ~110 kDa (Fig. 2b). Finally, we generated an eight-point mutant, CTNNBL1^L87R/I101R/G148R/Q192R/Q199R/Q238A/E278A/E282A^ (capping region, ARM I, ARM II, ARM III, and ARM IV). The mutant protein eluted in a monomeric state with a molecular weight of ~55 kDa in size-exclusion chromatography under 150 mM NaCl (Fig. 2a,b). In order to further investigate if the eight-point mutant CTNNBL1 exists as a monomer and maintains its conformation, SAXS measurements were performed (Supplementary Figs 1, 2, 3). The molecular weight of mutant was calculated as ~58 kDa, confirming the monomeric state of the mutant. We then reconstructed 3-D surface model of the mutant (Fig. 2c). When we compare the surface model of the mutant with the monomeric CTNNBL1 crystal structure by using the program SUPCOMB, the normalized spatial discrepancy (NSD) was 5.420. Considering that the NSD between surface model of the wild-type CTNNBL1 dimer without NaCl and the dimeric CTNNBL1 crystal structure was 4.423, we suspect that the mutant has a conformation similar to the monomeric CTNNBL1 crystal structure, although a little conformational change occurred in 150 mM NaCl. These results, combined with the results of the SAXS analysis described above, indicate that dimer formation is a unique feature of the CTNNBL1 protein and that the dimerization interface is at the large area formed by the N-terminal capping region and the first four ARM repeats.

### Characterization of the N-terminal domain of CDC5L

**Figure 3:**
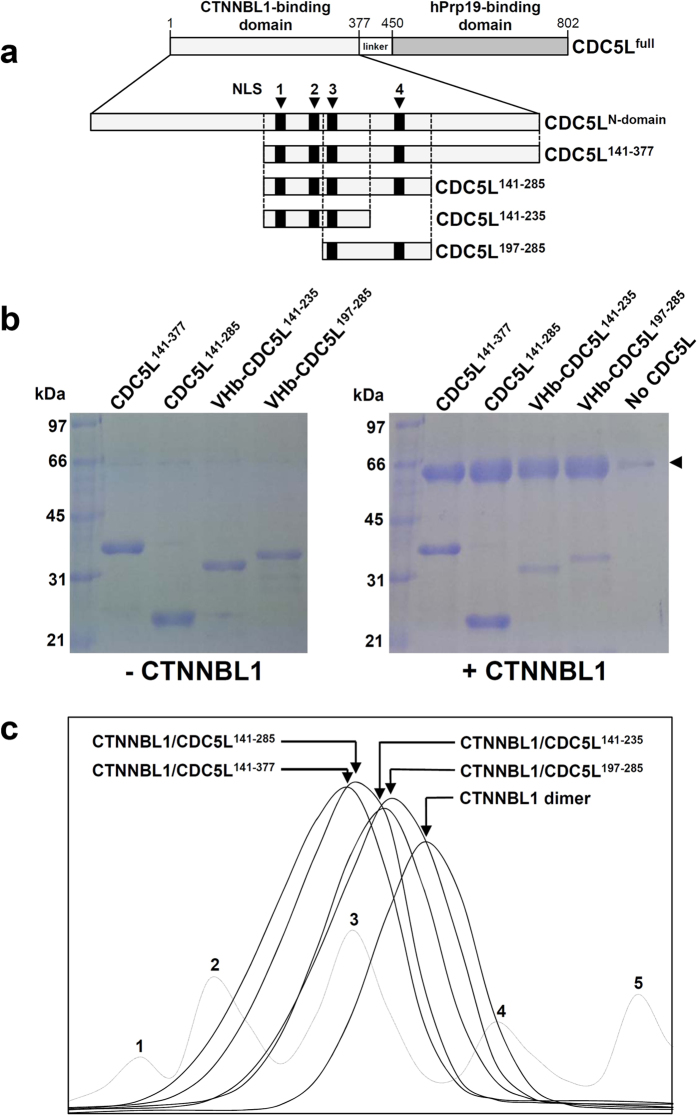
Interaction between CTNNBL1 and four truncated forms of CDC5L. (**a**) Construction of four truncated forms of CDC5L. The CTNNBL1- and hPrp19-binding domains of CDC5L are shown, and the residue numbers are indicated. The four NLS-like regions are shown as black boxes and labeled. The CDC5L^141–377^, CDC5L^141–285^, CDC5L^141–235^, and CDC5L^197–285^ constructs are shown as rectangular boxes. (**b**) Pull-down assays with CTNNBL1 and the four truncated forms of CDC5L. The left and right images show the SDS-PAGE patterns of the four truncated forms of CDC5L without and with CTNNBL1, respectively. The CDC5L proteins used are indicated at the top of each image. The molecular weights of the standard size markers are indicated on the left side of each image. CTNNBL1 is indicated with an arrow in the image on the right. (**c**) Size-exclusion chromatography analysis of the CTNNBL1/CDC5L complexes. The elution peaks of the CTNNBL1/CDC5L^141–377^, CTNNBL1/CDC5L^141–285^, CTNNBL1/CDC5L^141–235^, and CTNNBL1/CDC5L^197–285^ complexes and CTNNBL1 alone are indicated by arrows and labeled. For a precise analysis of molecular weight, standard samples of thyroglobulin (669 kDa), ferritin (440 kDa), aldolase (158 kDa), conalbumin (75 kDa), and ovalbumin (44 kDa) were used for calibration in size-exclusion chromatography experiments; the peaks are labeled from 1 to 5, respectively.

In the Prp19/CDC5L complex, CDC5L acts as a bridge connecting CTNNBL1 and Prp19 by binding to CTNNBL1 and Prp19 through its N-terminal domain (residues 1–377) and C-terminal domain (residues 450–802), respectively. We previously reported that CTNNBL1 uses its negatively-charged patch to interact with the N-terminal domain of CDC5L, which contains four NLS-like regions^18^. To investigate the interaction between CTNNBL1 and CDC5L in detail, we prepared several truncated forms of 6 × His-tagged CDC5L, including CDC5L^141–377^, CDC5L^141–285^, CDC5L^141–235^, and CDC5L^197–285^ (Fig. 3a). When we performed pull-down assays with Ni-NTA chromatography using these CDC5L constructs and CTNNBL1 without a 6xHis tag, all CDC5L proteins co-eluted with CTNNBL1 (Fig. 3b). Complex formation between CTNNBL1 and the truncated CDC5L proteins was confirmed in size-exclusion chromatography experiments (Fig. 3c). These results indicate that the overlapping region (residues 197–235) in the four CDC5L constructs is crucial for binding to CTNNBL1. Interestingly, this region of CDC5L contains the positively-charged NLS3 (residues 200–206: KKRKRKR). We suggest that the positively-charged NLS3-containing region (residues 197–235) binds to the negatively-charged patch of CTNNBL1, in the same way that importin-α recognizes nuclear localization sequences via charge-charge interactions.

Interestingly, we observed that two truncated forms of CDC5L, CDC5L^141–377^ and CDC5L^141–285^, migrated as dimers with molecular weights of ~64 and ~53 kDa, respectively, in size-exclusion chromatography (data not shown). Although we could not determine the oligomeric state of the two other CDC5L constructs, CDC5L^141–235^ and CDC5L^197–285^, because of their small sizes, we speculate that the N-terminal domain of CDC5L forms a homodimer using the region from residues 141 to 196.

### SAXS analysis of the CTNNBL1/CDC5L complex

**Figure 4:**
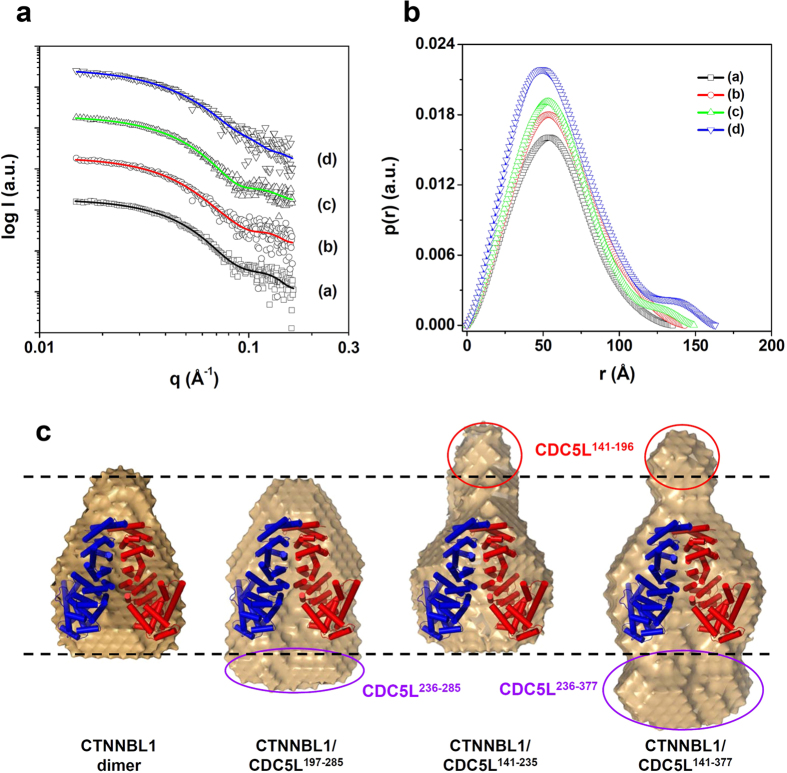
SAXS analysis of the CTNNBL1/CDC5L complexes. (**a**) X-ray scattering profiles of the CDC5L/CTNNBL1 complexes. The open symbols are the experimental data, and the solid lines are the X-ray scattering profiles obtained from the dummy atom models with the lowest χ = 1.560–1.680 values by the program DAMMIF. a–d are CTNNBL1 alone, CTNNBL1/CDC5L^197–285^, CTNNBL1/CDC5L^141–235^, and CTNNBL1/CDC5L^141–377^, respectively. For clarity, each plot is shifted along the log *I*(*q*) axis. (**b**) Pair distance distribution functions *p*(*r*) for CTNNBL1/CDC5L complexes calculated using experimental SAXS. The areas under the curves were normalized to the molecular weight. a–d are as described in (**a**). (**c**) Reconstructed structural models of the CTNNBL1/CDC5L complexes. For each reconstruction, five independent models were generated, compared, and averaged (Mean values of NSD of reconstructed models = 0.552–0.671). The filtered model was calculated using the program DAMAVER. For comparison of overall shapes and dimensions, the ribbon diagram of the atomic crystal models were superimposed onto the reconstructed dummy atoms models using the program SUPCOMB. (NSD = 4.423 for CTNNBL1 alone, 5.046 for CTNNBL1/CDC5L^197–285^, 5.070 for CTNNBL1/CDC5L^141–235^, 6.196 for CTNNBL1/CDC5L^141–377^) The CDC5L^141–196^ region located at the top of the N-terminal capping region of CTNNBL1 is indicated with a red circle and labeled. The CDC5L^236–285^ and CDC5L^236–377^ regions located at the bottom of ARM VII of CTNNBL1 are indicated with purple circles and labeled.

To determine a detailed structure of the CTNNBL1/CDC5L complex, we performed SAXS experiments using three CTNNBL1/CDC5L complexes containing CDC5L^141–377^, CDC5L^141–235^, and CDC5L^197–285^. The X-ray scattering profiles of the three complexes were similar to that of CTNNBL1 alone (Fig. 4a). In the *p*(*r*) function analysis, the patterns of the complexes and CTNNBL1 alone were similar (Fig. 4b). These results suggest that all three CTNNBL1/CDC5L complexes exist in a dimeric state with structures similar to that of CTNNBL1. However, a detailed *p*(*r*) function analysis showed that the *D*_max_ values of the CTNNBL1/CDC5L complexes were larger than that of CTNNBL1. They increased gradually in the order CTNNBL/CDC5L^197–285^ < CTNNBL1/CDC5L^141–235^ < CTNNBL1/CDC5L^141–377^, while the *R*_g,p(r)_ values of the complexes and CTNNBL1 were similar (Table 2). These results suggest that the length of the complex increases as the molecular weight of CDC5L increases (CDC5L^197–285^ < CDC5L^141–235^ < CDC5L^141–377^), while the thicknesses is maintained. Moreover, the *p*(*r*) function analysis of the complexes showed symmetric patterns as observed in CTNNBL1 (Fig. 4b). In light of the finding that CDC5L forms a homodimer, we propose that the CTNNBL1/CDC5L complex forms a heterotetramer consisting of one CTNNBL1 dimer and one CDC5L dimer.

Next, we reconstructed 3D structural models of the three CTNNBL1/CDC5L complexes. All three complexes had symmetric conformations and structures similar to that of CTNNBL1 alone. Moreover, the complexes were somewhat extended in the vertical direction when compared with CTNNBL1, which is consistent with the results of the *p*(*r*) function analysis (Fig. 4b). In order to identify the position of CDC5L in the CTNNBL1/CDC5L complex, we superposed the crystal structure of CTNNBL1 on the structural models of the three CTNNBL1/CDC5L complexes (Fig. 4c). In all three complexes, we identified a structural region where the CTNNBL1 dimer fit, and observed different parts protruding beyond CTNNBL1 depending on the complex. In the structural model of the CTNNBL1/CDC5L^141–377^ complex, protruding parts were observed at the top and bottom of CTNNBL1. In the structural models of CTNNBL1/CDC5L^141–235^ and CTNNBL1/CDC5L^197–285^, a protruding part was observed at the top and bottom of CTNNBL1, respectively. Interestingly, the protruding parts at the top of the CTNNBL1/CDC5L^141–377^ and the CTNNBL1/CDC5L^141–235^ complexes had similar structures. Moreover, the part protruding beyond CTNNBL1 at the bottom of the CTNNBL1/CDC5L^141–377^ complex was larger than that at the bottom of CTNNBL1/CDC5L^197–285^ complex (Fig. 4c). Considering that the NLS3-containing region of CDC5L (residues 197–235) binds to the ARM repeats of CTNNBL1, we suggest that CDC5L^141–196^ and CDC5L^236–377^ are located at the top and bottom of CTNNBL1, respectively.

## Discussion

We previously described the molecular architecture of the Prp19/CDC5L complex based on the dimeric form of CTNNBL1 observed in its crystal structure.^18^ CTNNBL1 is a key component of the Prp19/CDC5L complex, and elucidation of its oligomeric state is crucial for understanding the molecular architecture of the complex. In this study, we performed SAXS and site-directed mutagenesis experiments and showed that CTNNBL1 formed a dimer in physiological NaCl concentrations and its dimerization is similar to our dimeric CTNNBL1 structure. Based on these results, we propose that CTNNBL1 might function as a dimer in a cell. However, the dimerization interaction appeared to be weak: the CTNNBL1 dimer separated into two monomers when the NaCl concentration reached ~300 mM (data not shown). Moreover, other structural studies reported that CTNNBL1 existed as a monomer in crystal packing^5^,^19^. Considering that CTNNBL1 is involved in various cellular processes, such as mRNA splicing, DNA repair, protein degradation, transcriptional elongation, and immune-diversity^15^,^21^, we also suspect that CTNNBL1 can exist as a monomer depending on its cellular function.

We also investigated the binding of CDC5L to CTNNBL1 and the relative position of CDC5L in the CTNNBL1/CDC5L complex by performing SAXS experiments and confirmed the homo-dimerization of CDC5L N-terminus. From these data, we could conclude that CTNNBL1/CDC5L complex is the hetero-tetramer formed by the interaction between CTNNBL1 dimer and CDC5L dimer. This is well consistent with generating 3D model reconstructions of recombinant CTNNBL1 in complex with various CDC5L constructs. It has been well known that dimerization generally results to an enlarged interaction surface, which increases the potential for protein-protein interaction. Therefore, we conjecture that dimer-dimer interaction may contribute to recognize NLS3 of CDC5L preferably among four NLSs in CDC5L, which can confer the proper and rapid assembly in the formation of high dynamic and complicated hPrp19/CDC5L complex. It has not been possible to present a molecular mechanism of hPrp19/CDC5L complex in this study; the hetero-tetrameric architecture of CTNNBL1/CDC5L complex will contribute to uncover the molecular mechanism as a basic step to elucidate the overall structure and architecture of hPrp19/CDC5L complex.

## Methods

### Protein preparations

The gene coding region of human CTNNBL1 (residues 77–563) was amplified by polymerase chain reaction (PCR) from a human cDNA library and subcloned into a modified VHb fusion vector (pPosKJ) encoding a 6 × His tag and bacterial hemoglobin, which linked to the N-terminus of the target protein^22^. The resulting plasmid, pPosKJ:*ctnnbl1*, was transformed into the *Escherichia coli* BL21(DE3)-T1R strain (Sigma-Aldrich) and grown in LB medium. Protein overexpression was induced with 1.0 mM IPTG, and the cells were grown for 20 h at 18 °C. The cultured cells were harvested by centrifugation at 5000 × *g* for 20 min at 18 °C. The cell pellet was resuspended in ice-cold buffer A (50 mM Tris-HCl, pH 8.0) containing 10 mM imidazole and then disrupted by ultrasonication. The cell debris was removed by centrifugation at 11,000 × *g* for 1 h, and the lysate was bound to Ni-NTA agarose (Qiagen). The bound protein was washed with buffer A containing 20 mM imidazole and eluted with 300 mM imidazole in buffer A. The 6xHis tag and VHb were then removed by treatment with TEV protease (Invitrogen) followed by Ni-NTA chromatography. Trace amounts of contaminants were removed using size exclusion chromatography (Superdex200; GE Healthcare). The CTNNBL1 mutants were prepared through site-directed mutagenesis and purified following a procedure similar to that described for wild-type CTNNBL1. To produce two truncated forms of CDC5L, CDC5L^141–377^ and CDC5L^141–285^, the coding genes were subcloned into pET-30a(+). To produce the other two truncated forms of CDC5L, CDC5L^141–235^ and CDC5L^197–285^, the coding genes were subcloned into the pPosKJ expression vector. The truncated forms of CDC5L were purified following a procedure similar to that used with CTNNBL1. However, 300 mM NaCl was added to the buffers throughout the purification procedure to avoid precipitation of CDC5L, and the 6xHis-tagged proteins were prepared without treatment with TEV protease.

### Small-angle X-ray scattering data acquisition and processing

SAXS measurements were carried out at the In-vacuum Undulator 20 beamline (4C SAXS II) of the Pohang Accelerator Laboratory (PAL) Korea. The wavelength and beam size were 0.675 Å and 0.2 (V) × 0.6 (H) mm^2^, respectively. A two-dimensional charge-coupled detector (Mar USA, Inc.) was employed. A sample-to-detector distance of 4.00 m for SAXS was used. The magnitude of the scattering vector, *q* = (4*π/λ*) sin *θ*, was 0.010 Å^−1^ < *q* < 0.165 Å^−1^, where 2*θ* is the scattering angle and is the wavelength of the X-ray beam source. The scattering angle was calibrated with polyethylene-*b*-polybutadiene-*b*-polystyrene (SEBS) block copolymer standard. We used solution sample cells with 10-μm-thick mica windows, a volume of 50 μl, and an X-ray beam path length of 0.8 mm. All scattering measurements were carried out at 4 °C. The SAXS data were collected in five successive frames of 0.1 min each to monitor radiation damage. There were no changes in the scattering patterns with time, i.e., no radiation damage was detected during the scattering measurements. Protein solutions were measured over a small concentration range, 1.0–5.0 mg/ml, in order to obtain good quality scattering data without any interference from protein molecules (i.e., concentration effect). A low concentration range of 1.0–2.0 mg/ml provided high quality scattering data without concentration effect. Each 2D SAXS pattern was circularly averaged from the beam center and normalized to the transmitted X-ray beam intensity, which was monitored with a scintillation counter placed behind the sample. In the case of CTNNBL1, the measurements were taken in solutions with 0, 100, 200, 300, 400, and 500 mM NaCl, after using the solutions as the experiment background. The measurement of mutant were performed in solutions with 100 mM NaCl, after using the solutions as the experiment background. The measured concentrations of the CTNNBL1/CDC5L construct solutions were 1.0–2.0 mg/ml. The scattering of a 40 mM Tris-HCl pH 8.0 buffer solution without NaCl was used as the experimental background to minimize the conformational change effect by NaCl. The *R*_g,G_ (radius of gyration) and forward scattering intensity *I*(0) at zero angle values were estimated from the scattering data using Guinier analysis^23^. The molecular mass (MM) was calculated from the known scattering of water of 0.01670 cm^−1^ at 4 °C.^24^ The pair distance distribution *p*(*r*) function was obtained through the indirect Fourier transform method using the program GNOM^25^.

### Construction of 3D structural models

To reconstruct the molecular shapes, the ab initio shape determination program DAMMIF^26^ was used. For each model reconstruction, five independent models were selected, and the averaged aligned model was filtered at a given cutoff volume using the program DAMAVER^27^. The final models were obtained by imposing P2 symmetry restriction. The SAXS curves were calculated from the atomic models using the program CRYSOL^28^. For comparison of the overall shapes and dimensions, the ribbon diagrams of the atomic crystal models were superimposed onto the reconstructed dummy atom models using the program SUPCOMB^29^.

### Pull-down assays

Each truncated form of the 6 × His-tagged CDC5L protein was mixed with the native CTNNBL1 protein in 50 mM Tris-HCl, pH 8.0 and 300 mM NaCl. The protein mixture was dialyzed against 50 mM Tris-HCl without NaCl at 4 °C overnight and then loaded onto a Ni-NTA resin. The bound protein was washed with a buffer containing 50 mM Tris-HCl, pH 8.0 and 10 mM imidazole and then eluted with a buffer containing 50 mM Tris-HCl, pH 8.0 and 300 mM imidazole. CTNNBL1 and CDC5L complex formation was confirmed with SDS-PAGE.

### Size-exclusion chromatographic analysis

In order to measure the molecular weight of proteins, 500 μl 2mg/ml protein was injected onto a Superdex 200 10/300 column (GE Healthcare) at a flow rate of 0.5 ml/min at 40 mM Tris-HCl, pH 8.0, 150 mM NaCl. The wild type CTNNBL1 was monitored using buffer containing 400 mM NaCl to present monomeric state as a control. The molecular weights of the eluted samples were calculated based on the calibration curve of standard samples. The molecular weight of CTNNBL1/CDC5L complex was calculated as similar method by using 500 μl 4 mg/ml protein mixture with 1:1 ratio, and co-elution by the formation of CTNNBL1/CDC5L complex was evaluated by SDS-PAGE.

## Additional Information

**How to cite this article**: Ahn, J.-W. *et al.* Small angle X-ray scattering studies of CTNNBL1 dimerization and CTNNBL1/CDC5L complex. *Sci. Rep.* **5**, 14251; doi: 10.1038/srep14251 (2015).

## Supplementary Material

Supplementary Information

## Figures and Tables

**Table 1:** Structural parameters obtained from the SAXS data of CTNNBL1 in various NaCl concentrations.

**NaCl (mM)**	***R*_g,G_**^a^ **(Å)**	***R*_g,p(r)_**^b^ **(Å)**	***D*_max_**^c^ **(Å)**	***I*(0)_Abs_**^d^	**MM**^e^ **(kDa)**	**Conformation**
Crystal	34.89 ± 0.008	34.87 ± 0.018	106.0	ND^f^	110	dimer
0	41.80 ± 0.385	42.97 ± 0.140	135.0	0.1466	101	dimer
100	44.20 ± 0.541	45.73 ± 0.165	146.9	0.1834	126	dimer
200	46.40 ± 0.752	49.53 ± 0.221	163.5	0.1604	110	dimer
mutant^f^	42.11 ± 1.850	43.94 ± 2.310	163	0.0841	58	monomer

^a^*R*_g,G_ (radius of gyration) was obtained from the scattering data by the Guinier analysis.

^b^*R*_g,p(r)_ (radius of gyration) was obtained from the *p*(*r*) function by the program GNOM.

^c^*D*_max_ (maximum dimension) were obtained from the *p*(*r*) function by the program GNOM.

^d^*I*(0) (forward scattering intensity) was obtained from the scattering data by the Guinier analysis.

^e^MM (molecular mass) was estimated from absolute *I*(0) intensity.

^f^Mutant stands for CTNNBL1^L87R/I101R/G148R/Q192R/Q199R/Q238A/E278A/E282A^.

**Table 2:** Structural parameters obtained from the SAXS data of CDC5L/CTNNBL1 complexes.

**CDC5L/CTNNBL1 complexes**	***R*_g,G_**^a^ **(Å)**	***R*_g,p(r)_**^b^ **(Å)**	***D*_max_**^c^ **(Å)**	**Abs *I*(0)/c**^d^	**MM**^e^ **(kDa)**
CTNNBL	41.80 ± 0.385	42.97 ± 0.140	135.0	0.0814	101
CTNNBL/CDC5L^197–285^	43.40 ± 0.481	45.44 ± 0.206	143.0	0.0913	113
CTNNBL1/CDC5L^141–235^	43.70 ± 0.439	44.75 ± 0.204	149.0	0.0956	118
CTNNBL1/CDC5L^141–377^	46.30 ± 0.824	47.16 ± 0.357	163.0	0.1140	141

^a^*R*_g,G_ (radius of gyration) was obtained from the scattering data by the Guinier analysis.

^b^*R*_g,p(r)_ (radius of gyration) was obtained from the *p*(*r*) function by the program GNOM.

^c^*D*_max_ (maximum dimension) was obtained from the *p*(*r*) function by the program GNOM.

^d^*I*(0)/c (forward scattering intensity/concentration) was obtained from the scattering data by the Guinier analysis.

^e^MM (molecular mass) was estimated from absolute *I*(0) intensity.
