# Supplementary material for: Small angle X-ray scattering studies of CTNNBL1 dimerization and CTNNBL1/CDC5L complex
Source: Sci Rep. 2015 Sep 18;5:14251. doi: 10.1038/srep14251 (PMC4585563; doi:10.1038/srep14251)
Supplement: Supplementary Information [file srep14251-s1.doc]

Supplementary Materials

**Small angle X-ray scattering studies of CTNNBL1 dimerization and CTNNBL1/CDC5L complex**

Jae-Woo Ahn, Kyeong Sik Jin, Hyeoncheol Francis Son, Jeong Ho Chang and Kyung-Jin Kim

*Correspondence should be addressed to: Email: [kkim@knu.ac.kr](mailto:kkim@knu.ac.kr)

Supplementary Figures 1 to 3.

**Supplementary Figure 1.** X-ray scattering profiles of the 8-point CTNNBL1 mutant in 150 mM NaCl solution. The open symbols are experimental data and the solid lines are the X-ray scattering profiles obtained from the dummy atoms models with the lowest χ^2^ = 0.04–0.3 values by the program DAMMIF. For clarity, plot is shifted along the log *I(q)* axis.

**Supplementary Figure 2.** Guinier plots of the X-ray scattering profiles of the 8-point CTNNBL1 mutant in 150 mM NaCl solution. The straight lines were obtained from the linear regression of the scattering data in the *q*^2^ region. For clarity, plot is shifted along the ln *I*(*q*) axis.

**Supplementary Figure 3.** Pair distance distribution functions *p*(*r*) for the 8-point CTNNBL1 mutant in 150 mM NaCl solution. The *p*(*r*) functions in solution were calculated using the program GNOM.
